# ADESTI: A portable open source instrument for air quality monitoring in surgical operating rooms

**DOI:** 10.1016/j.ohx.2026.e00818

**Published:** 2026-07-26

**Authors:** Prisma Megantoro, Anna Surgean Veterini, Nagesparan Ainarappan, Nayu Nurrohma Hidayat, Fikarul Mujtahida, Mochammad Dava Septiadi Pratama, Muhammad Aqil Afham Rahmat

**Affiliations:** a Faculty of Advanced Technology and Multidiscipline, Universitas Airlangga, Jl. Dr. Ir. H. Soekarno, Mulyorejo, Surabaya, East Java 60115, Indonesia; b Department of Anesthesiology and Reanimation, Faculty of Medicine, Universitas Airlangga, Jl. Mayjend Prof. Dr. Moestopo, No. 47, Surabaya, East Java 60131, Indonesia; c Department of Anesthesiology and Reanimation, Dr. Soetomo General Academic Hospital, Jl. Mayjend Prof. Dr. Moestopo, No. 6-8, Surabaya, East Java 60286, Indonesia; d Department of Electrical and Electronics Engineering, Faculty of Engineering, Technology and Built Environment, UCSI University, 1 Jalan UCSI, UCSI Heights, Taman Connaught, Cheras, 56000 Kuala Lumpur, Malaysia; e Department of Mechanical and Manufacturing Engineering, Faculty of Engineering, Universiti Putra Malaysia, 43400 UPM Serdang, Selangor, Malaysia

**Keywords:** Operating room air quality, Environmental gas monitoring, Good health and well-being, Arduino embedded system, Open-source hardware

## Abstract

This paper presents the design and development of the Air Quality Detection and Safety-Targeted Instrument (ADESTI), a portable smart instrument for monitoring air quality in surgical operating rooms. The system is motivated by the need to detect deviations in oxygen concentration. Such deviations can indicate gas supply leakage or ventilation failure, which are safety concerns during surgical and anesthesia procedures. ADESTI integrates three gas sensors, namely an electrochemical oxygen sensor, a Non-Dispersive Infrared (NDIR) carbon dioxide sensor, and an electrochemical carbon monoxide sensor. It also includes an environmental sensor for temperature, humidity, barometric pressure, and light intensity. All sensors interface with an Arduino Mega 2560 R3 embedded system through the I^2^C and UART protocols for real-time data acquisition. The measured values are shown on an integrated Thin-Film Transistor (TFT) display. The enclosure is fabricated from Polylactic Acid Plus (PLA + ) by 3D printing to produce a compact and durable casing. The firmware, developed in the Arduino IDE, handles sensor initialization and real-time data acquisition and display. Characterization against reference instruments showed accuracies of 99.71% for O_2_, 98.84% for CO_2_, and 93.79% for CO, with precision above 99% for all three gas sensors. A key feature of ADESTI is that it combines the functions of four separate commercial instruments into a single low-cost and open-source device. It was field-validated across various surgical procedures at General Academic Hospital, Surabaya, Indonesia, at a total hardware cost of 355.10 USD. All design files, firmware, and the bill of materials are openly available at https://doi.org/10.17632/b6mgd6fgth.1.

## Highlights

• Portable open-source instrument for operating room air quality monitoring.
• Integrates O2, CO, CO2, and environmental sensors on Arduino Mega 2560 R3.
• Real-time multi-parameter display on a TFT screen in a 3D-printed enclosure.
• Achieves 99.71% O2, 98.84% CO2, and 93.79% CO accuracy versus reference.
• Field-validated at RSUD Dr. Soetomo across multiple surgical procedures.

## Graphical abstract

**Figure:**
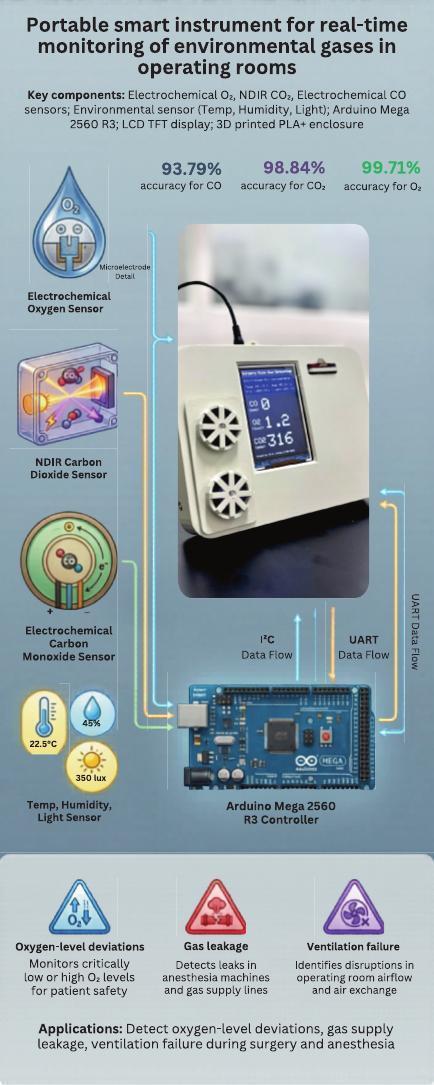

Specifications table

**Table:** 

Hardware name	ADESTI
Subject area	• Biomedical engineering • Instrumentation
Hardware type	• Field measurements and sensors
Closest commercial analog	• PO_2_-250 (O_2_) • GC-2028 (CO_2_) • GCO-2008 (CO) • MHB-382SD (temperature and humidity)
Open source license	• Hardware: CERN-OHL-S v2.0 • Software: MIT License • Documentation: CC BY 4.0
Cost of hardware	355.10 USD
Source file repository	https://doi.org/10.17632/b6mgd6fgth.1

## 1 Hardware in context

The operating room presents a range of environmental hazards for the surgical team, including noise, distractions, and airborne exposures [1].This room is one of the most complex environments within a hospital, where environmental quality control is essential [2]. Surgical performance and patient outcomes depend on how well the room is organized and regulated [3], [4]. Rigorous regulation must be maintained over several factors, including air quality, temperature, humidity, and ventilation, all of which contribute to patient safety and to the prevention of contamination [5]. Among these factors, the composition of the room air is particularly important, because deviations in gas concentrations can indicate hazards that affect both patients and the surgical team. One well-documented concern is the potential exposure to anaesthetic gases, which, although administered with care, may leak into the surrounding environment [6], [7]. Such leakage has been observed in clinical practice [8] and represents a recognized occupational concern [9].

### 1.1 Clinical background and occupational exposure

General anesthesia is the reversible loss of consciousness and of the ability to respond to noxious stimuli [10]. It involves complex effects on the central nervous system [9] and carries perioperative neurocognitive considerations [11]. Anesthetic agents induce unconsciousness, analgesia, amnesia, and muscle relaxation in patients undergoing invasive procedures [12], [13], and the anesthetic state is closely linked to the management of the surgical stress response [14]. Inhalational agents such as nitrous oxide, sevoflurane, desflurane, and isoflurane are predominantly used for their rapid onset and stable anesthetic state [15], [16]. These volatile agents act on the central nervous system to maintain unconsciousness, analgesia, and muscle relaxation [17], [18]. The choice of agent depends on the surgical procedure and the patient's condition [19], on the need for swift postoperative recovery [20], and on the recommendation to minimize unnecessary exposure [21].

Although administered with care, these agents may adversely affect the healthcare personnel involved [22], so balancing patient needs with occupational impact has become an important clinical consideration [23], [24]. The harmful effects of occupational exposure have been documented through human biomonitoring studies [25] and occupational health reviews [26]. Elevated anesthetic gas levels and insufficient evacuation systems are significant contributors to high anesthetic residues [27], and the environmental implications of these gases have also been highlighted [28]. Both short term and long term health effects have been reported [29]. A study at a Brazilian university hospital linked occupational exposure in theaters lacking scavenging systems to headaches, irritability, neurobehavioral alterations, and DNA damage [30]. Waste anesthetic gases, including N_2_O and halogenated agents, are frequently released into surgical and recovery room air, exposing doctors, nurses, and surgical technicians [31], [32].

### 1.2 Existing monitoring approaches

Beyond waste anesthetic gases, the general gas composition of the operating room air is itself an indicator of safety and ventilation performance. Room oxygen concentration reflects the integrity of the gas supply and the adequacy of ventilation, so a deviation may signal a leak or a ventilation failure. Carbon dioxide accumulation indicates insufficient air exchange. Carbon monoxide serves as a marker of environmental contamination. At present, air quality in the operating room is managed mainly through scavenging systems and general ventilation. Gas monitoring systems have been explored as a complementary measure. These include smart gas leakage monitoring for hospitals [33], portable sensor node detectors [34], and IoT based gas detection systems [35]. Monitoring the three gases together with temperature, humidity, pressure, and light provides a practical real time picture of operating room air quality during anesthesia procedures. The air quality of the operating room has long been recognized as an important factor for both patient and staff safety [36]. Beyond anesthetic gases, other airborne contaminants such as surgical smoke have also been identified as hazards in the operating room [37]. Centralized and automated monitoring systems for medical gases in hospitals have also been proposed [38], [39].

### 1.3 Comparison with existing systems and novelty of ADESTI

A dedicated comparison in Table 1 clarifies how ADESTI differs from existing systems. Most prior gas monitoring systems target either general gas leak detection or broad indoor air quality. They are not specific to the operating room environment. Several are single gas systems, and most are not validated inside an actual surgical setting. The systems by Hussien et al., Shukur and Al-adilee, and Pillco-Sanchez et al. focus on leak detection for hospitals, general spaces, or residential kitchens. None of them measures oxygen, carbon dioxide, and carbon monoxide together as combined indicators of operating room air quality and ventilation performance.

**Table 1:** Comparison of ADESTI with existing gas monitoring systems and commercial instruments.

**System / Study**	**Target application**	**Parameters measured**	**Platform**	**Open source**	**Field validated in OR**
ADESTI (this work)	Operating room air quality	O_2_, CO_2_, CO, temperature, humidity, pressure, light	Arduino Mega 2560	Yes	Yes (General Academic Hospital Dr. Soetomo)
Hussien et al. [32]	Hospital gas leakage	Single gas leakage indicator	IoT node	No	No
Shukur & Al-adilee [33]	General portable gas leak	Gas leak (off the shelf node)	IoT node	No	No
Pillco-Sanchez et al. [34]	Residential kitchen gas leak	Natural gas leak	IoT node	No	No
Commercial units (Lutron series)	General IAQ / single parameter	One or two parameters per unit	Proprietary handheld	No	No

Commercial instruments cover these parameters, but only in a fragmented way. Each Lutron reference meter measures one or two parameters at a time. Covering the full parameter set of ADESTI would require four separate handheld instruments. None of these commercial units is open source, and none is designed for continuous multi parameter monitoring inside the operating room.

The novelty of ADESTI lies in this integration. It combines oxygen, carbon dioxide, and carbon monoxide sensing with temperature, humidity, pressure, and light into a single low cost and open source platform. It was designed specifically for the operating room use case. It was also field validated across real surgical procedures at General Academic Hospital Dr. Soetomo.

Several limitations of the current design are acknowledged. First, the electrochemical CO and O_2_ sensors retain some residual cross sensitivity to other reducing or oxidizing gases that were not evaluated in this study. Second, the current firmware does not implement onboard data logging or wireless data transmission. All measurements are displayed in real time but not automatically stored. Third, field deployment revealed a persistent offset in O_2_ readings relative to laboratory calibration conditions. This offset is discussed in Section 7.4.

## 2 Hardware description

The ADESTI system was developed as a portable environmental monitoring device based on the Arduino Mega 2560 R3 microcontroller. The Arduino Mega 2560 R3 was selected because it provides a large number of GPIO pins, supports multiple hardware serial ports for several UART based sensors at once, offers sufficient SRAM for concurrent sensor handling, and is compatible with widely available sensor modules. These features are important because ADESTI operates several sensors in parallel over both I^2^C and UART, which requires multiple serial channels and stable memory handling. All gas and environmental sensors used in ADESTI provide digital outputs through I^2^C or UART. For this reason, no external analog signal conditioning, filtering, or amplification is required between the sensors and the microcontroller, since the analog to digital conversion and internal compensation are handled inside each sensor module.

The instrument casing was fabricated from matte PLA + material by 3D printing using fused deposition modeling. The detailed print settings are provided in the Build instructions.

The gas sensors require a short warm up period after power on before the readings stabilize. The oxygen and carbon monoxide sensors are factory calibrated, and the calibration procedure applied before field deployment is described in detail in Section 7.

The sensors communicate with the microcontroller through two interfaces. The oxygen sensor, the carbon monoxide sensor, and the environmental sensor use the I^2^C interface at the standard 100 kHz clock, with the oxygen and carbon monoxide sensors assigned to addresses 0x75 and 0x74. The carbon dioxide sensor uses a UART interface at a baud rate of 9600 through a command and response protocol. The oxygen and carbon monoxide sensors operate in passive acquisition mode with temperature compensation enabled. The firmware reads all sensors and refreshes the display once every 2 s.

**Fig. 1:**
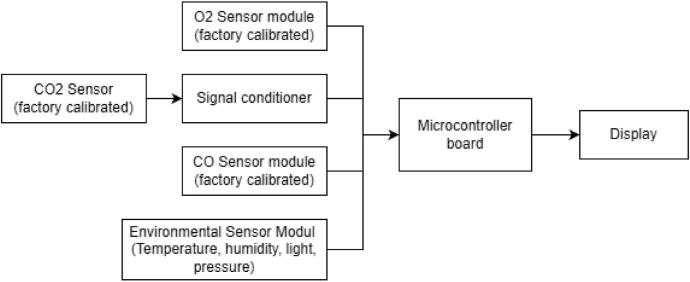
Block diagram of the ADESTI system showing the connection between the sensors and the Arduino Mega 2560 R3 microcontroller.

Fig. 1 shows the block diagram of the ADESTI system. The oxygen sensor, the carbon monoxide sensor, and the carbon dioxide sensor are connected to the onboard microcontroller. The temperature, humidity, barometric pressure, and ambient light parameters are measured by a single environmental sensor module, which is also connected to the microcontroller. The Arduino Mega 2560 R3 acts as the central controller. It reads all sensor data and displays the measured values on the LCD display. The oxygen, carbon monoxide, and environmental sensors communicate through the I^2^C interface, while the carbon dioxide sensor communicates through the UART interface.

The selection of each sensor was guided by the combination of measurement principle, cross-sensitivity behavior, response time, and power consumption relevant to the enclosed and chemically complex OR environment, as summarized in Table 2.

**Table 2:** Specification of the sensors used for ADESTI.

**Sensor**	**Model**	**Sensing Principle**	**Range**	**Resolution**	**Response Time**	**Power (5 V)**
O_2_	SEN0465	Electrochemical	0–25 %Vol	0.1 %Vol	≤15 s (T90)	<5 mA
CO	SEN0466	Electrochemical	0–1000 ppm	1 ppm	≤30 s (T90)	<5 mA
CO_2_	SEN0220	NDIR	0–50000 ppm	−	<30 s (T90)	<85 mA
Temperature/ Humidity	SEN0501 (SHTC3)	Capacitive/ thermal	−40–125 °C / 0–100% RH	0.01 °C / 0.01% RH	−	shared
Barometric pressure	SEN0501 (BMP280)	Piezoresistive MEMS	300–1100 hPa	−	−	shared
Ambient light	SEN0501 (VEML7700)	Photodiode	0–120 klux	0.0036 lx/ct	−	shared

For O_2_ and CO, an electrochemical sensing principle (SEN0465, SEN0466) was selected over metal-oxide semiconductor (MOS) alternatives namely the MQ-series. MOS sensors require a continuous heating element to maintain sensitivity, exhibit higher baseline drift, and are more prone to cross-sensitivity toward volatile anesthetic agents (e.g., sevoflurane, isoflurane) present in the OR air. Electrochemical sensors operate at ambient temperature, draw under 5 mA, and offer better gas selectivity. It is important given the mixed-gas background of a surgical environment. Their response time (T90 ≤ 15 s for O_2_, ≤30 s for CO) is adequate for early leak/ventilation-failure detection rather than millisecond-level transient tracking.

For CO_2_, a non-dispersive infrared (NDIR) sensor (SEN0220) was chosen over electrochemical or MOS CO_2_ alternatives because NDIR measurement is independent of ambient oxygen concentration. It is necessary given that O_2_ itself is a monitored, fluctuating variable in this application. NDIR sensors are also immune to poisoning by other gases and offer a stable long-term range (0–50000 ppm), consistent with their longer service life and reducing replacement frequency during repeated field deployment.

The SEN0501 module was selected for temperature, humidity, barometric pressure, and ambient light because it integrates all four parameters on a single I^2^C/UART interface avoiding the wiring and firmware overhead of separate discrete sensors. Combined, the four active sensing modules draw approximately 140 mA at 5 V (≈0.7 W), a power budget compatible with the USB-powered operation of the Arduino Mega 2560 R3. The usefulness of this hardware for other researchers:

• Integrated multi parameter monitoring. The device measures O_2_, CO_2_, and CO together with temperature, humidity, pressure, and light in a single portable instrument.
• Open source and modifiable. The Arduino IDE based firmware and open hardware design allow other researchers to add sensors, change threshold parameters, or integrate wireless transmission.
• Low cost and replicable. The device uses widely available modules and can be reproduced at a much lower cost than comparable commercial instruments.

## 3 Design files summary

All design files for ADESTI are openly available in the Mendeley Data repository at https://doi.org/10.17632/b6mgd6fgth.1, which supports file versioning. These files include the firmware, the editable SketchUp enclosure source model, the print-ready STL files, the wiring schematics, the bill of materials, and the photographic documentation. They provide everything required to reproduce the ADESTI hardware. All files are released under the licenses indicated in the table below. A short description of each file is provided after the summary Table 3.

**Table 3:** Design files summary.

**Design file name**	**File type**	**Open source license**	**Location of the file**
ADESTI_Firmware_v4.2	Arduino firmware source code (.ino)	MIT License	Available in the Mendeley Data repository
ADESTI_Enclosure_Source_rev1	SketchUp 3D model (.skp)	CERN-OHL	Available in the Mendeley Data repository
ADESTI_Enclosure_Print_caseTop_rev1	3D printing file (.stl)	CERN-OHL	Available in the Mendeley Data repository
ADESTI_Enclosure_Print_caseBottom	3D printing file (.stl)	CERN-OHL	Available in the Mendeley Data repository
ADESTI_Enclosure_Print_coverSensor	3D printing file (.stl)	CERN-OHL	Available in the Mendeley Data repository
ADESTI_Enclosure_Print_stand	3D printing file (.stl)	CERN-OHLba	Available in the Mendeley Data repository
ADESTI_Block_Diagram	Wiring schematic (.pdf)	CC BY 4.0	Available in the Mendeley Data repository
ADESTI_Detailed_Schematic	Wiring schematic (.pdf)	CC BY 4.0	Available in the Mendeley Data repository
ADESTI_Bill_of_Material	Bill of materials (.pdf)	CC BY 4.0	Available in the Mendeley Data repository
ADESTI_Documentation_Photos1	Image file (.jpg)	CC BY 4.0	Available in the Mendeley Data repository
ADESTI_Documentation_Photos2	Image file (.jpg)	CC BY 4.0	Available in the Mendeley Data repository
ADESTI_Documentation_Photos3	Image file (.jpg)	CC BY 4.0	Available in the Mendeley Data repository

For each design file listed in the summary table above, a short description of the file is included below.

• ADESTI_Firmware_v4.2. Arduino IDE firmware written in C++ for the Arduino Mega 2560 R3. It handles sensor initialization, multi-sensor data acquisition over I^2^C and UART, TFT display visualization, and the safety threshold logic for continuous environmental monitoring.
• ADESTI_Enclosure_Source_rev1. Editable SketchUp 3D source model of the ADESTI enclosure. The model includes component mounting positions, ventilation openings, and cable routing channels, and can be modified to accommodate additional sensors or layout changes.
• ADESTI_Enclosure_Print_caseTop_rev1. Print-ready STL file of the top part of the ADESTI enclosure, exported from the SketchUp source model for fused deposition modeling 3D printing in PLA + .
• ADESTI_Enclosure_Print_caseBottom. Print-ready STL file of the bottom part of the ADESTI enclosure, exported from the SketchUp source model for fused deposition modeling 3D printing in PLA + .
• ADESTI_Enclosure_Print_coverSensor. Print-ready STL file of the sensor cover component of the ADESTI enclosure, providing mechanical protection while allowing gas exposure to the sensors.
• ADESTI_Enclosure_Print_stand. Print-ready STL file of the supporting stand for the ADESTI enclosure, used to position the instrument stably during operation.
• ADESTI_Block_Diagram. Block-level wiring schematic of the ADESTI hardware, showing the connections between the Arduino Mega 2560 R3, the gas and environmental sensors, the TFT display, and the power adapter.
• ADESTI_Detailed_Schematic. Detailed pin-level wiring schematic of the ADESTI hardware, showing specific Arduino Mega 2560 R3 pin assignments, the I^2^C and UART connections, and the 5 V power routing through the DFRobot I^2^C hub.
• ADESTI_Bill_of_Material. Bill of materials listing all components required to build the ADESTI hardware, with the designator, quantity, unit and total cost, source of materials, and material type for each component.
• ADESTI_Documentation_Photos1. Photographic documentation of the assembled ADESTI instrument, showing the external enclosure and overall hardware.
• ADESTI_Documentation_Photos2. Photographic documentation of the ADESTI hardware, showing the internal component layout and sensor placement.
• ADESTI_Documentation_Photos3. Photographic documentation of the ADESTI hardware, showing the wiring arrangement and assembly details.

## 4 Bill of materials summary

The bill of materials summary lists in Table 4 all components required to build the ADESTI hardware, including the microcontroller, gas and environmental sensors, display, power supply, and enclosure material. For each component, the table provides the designator, quantity, unit and total cost, source of materials, and material type. The designators correspond to the component labels used in the wiring schematic.

**Table 4:** Bill of material summary.

**Designator**	**Component**	**Number**	**Cost per unit −currency**	**Total cost −** **currency**	**Source of materials**	**Material type**
U1	Arduino Mega 2560 R3 microcontroller	1	45.00 USD	45.00 USD	store.arduino.cc	Composite
S1	Electrochemical O_2_ sensor (SEN0465)	1	84.90 USD	84.90 USD	dfrobot.com	Semiconductor
S2	Electrochemical CO sensor (SEN0466)	1	66.90 USD	66.90 USD	dfrobot.com	Semiconductor
S3	NDIR CO_2_ sensor (SEN0220)	1	89.00 USD	89.00 USD	dfrobot.com	Semiconductor
S4	Environmental sensor (SEN0501)	1	29.90 USD	29.90 USD	dfrobot.com	Semiconductor
D1	3.5″ TFT LCD display shield	1	15.00 USD	15.00 USD	Commercial supplier	Composite
H1	DFRobot I^2^C hub	1	5.90 USD	5.90 USD	dfrobot.com	Composite
W1	Gravity 4-pin connector cable set	1	5.00 USD	5.00 USD	dfrobot.com	Composite
P1	12 V / 5 A AC-DC power adapter	1	10.00 USD	10.00 USD	Commercial supplier	Composite
E1	PLA + filament for enclosure (∼150 g)	1	3.50 USD	3.50 USD	Commercial supplier	Polymer

Note: Unit prices are approximate values from DFRobot and international marketplaces and may vary by supplier and region.

## 5 Build instructions

This section describes the step-by-step construction of the ADESTI hardware, from component preparation to final assembly verification. The instructions are intended to allow other researchers to reproduce the instrument using widely available commercial sensor modules and a standard Arduino platform. The hardware was designed with a modular approach, so each sensor module can be assembled, replaced, or expanded independently without redesigning the entire system. All required components, design files, and the firmware are listed in the Bill of materials and Design files summary.

The construction of the ADESTI hardware begins with the preparation of all electronic component. These include the Arduino Mega 2560 R3 microcontroller, the O_2_ sensor (SEN0465), the CO sensor (SEN0466), the CO_2_ sensor (SEN0220), the SEN0501 environmental sensor, the TFT display, the DFRobot I^2^C hub, jumper cables, and a 12 V/5 A power adapter. The hardware architecture was designed using a modular approach to simplify assembly, maintenance, and future sensor expansion. The Arduino Mega 2560 R3 was selected because it supports multiple communication protocols namely UART and I^2^C while providing sufficient GPIO pins for multi-sensor integration. Initially, all sensors were tested individually using the Arduino IDE to verify operational functionality and communication compatibility before full system integration.

The electronic assembly takes approximately 2 to 4 h, excluding the enclosure printing time. The assembly process starts by connecting the gas and environmental sensors to the DFRobot I^2^C hub and the UART interface. The O_2_ sensor, the CO sensor, and the SEN0501 environmental sensor are connected through the shared I^2^C bus on pins 20 (SDA) and 21 (SCL). Each sensor using a unique address to avoid communication conflicts, with the O_2_ sensor at 0x75 and the CO sensor at 0x74. The CO_2_ sensor uses UART serial communication on pins 53 (RX) and 51 (TX). Proper cable management and stable wiring layouts are necessary to minimize electrical noise and prevent unstable readings during long-term operation. The TFT display is then connected to the microcontroller to provide real-time visualization of sensor outputs.

**Fig. 2:**
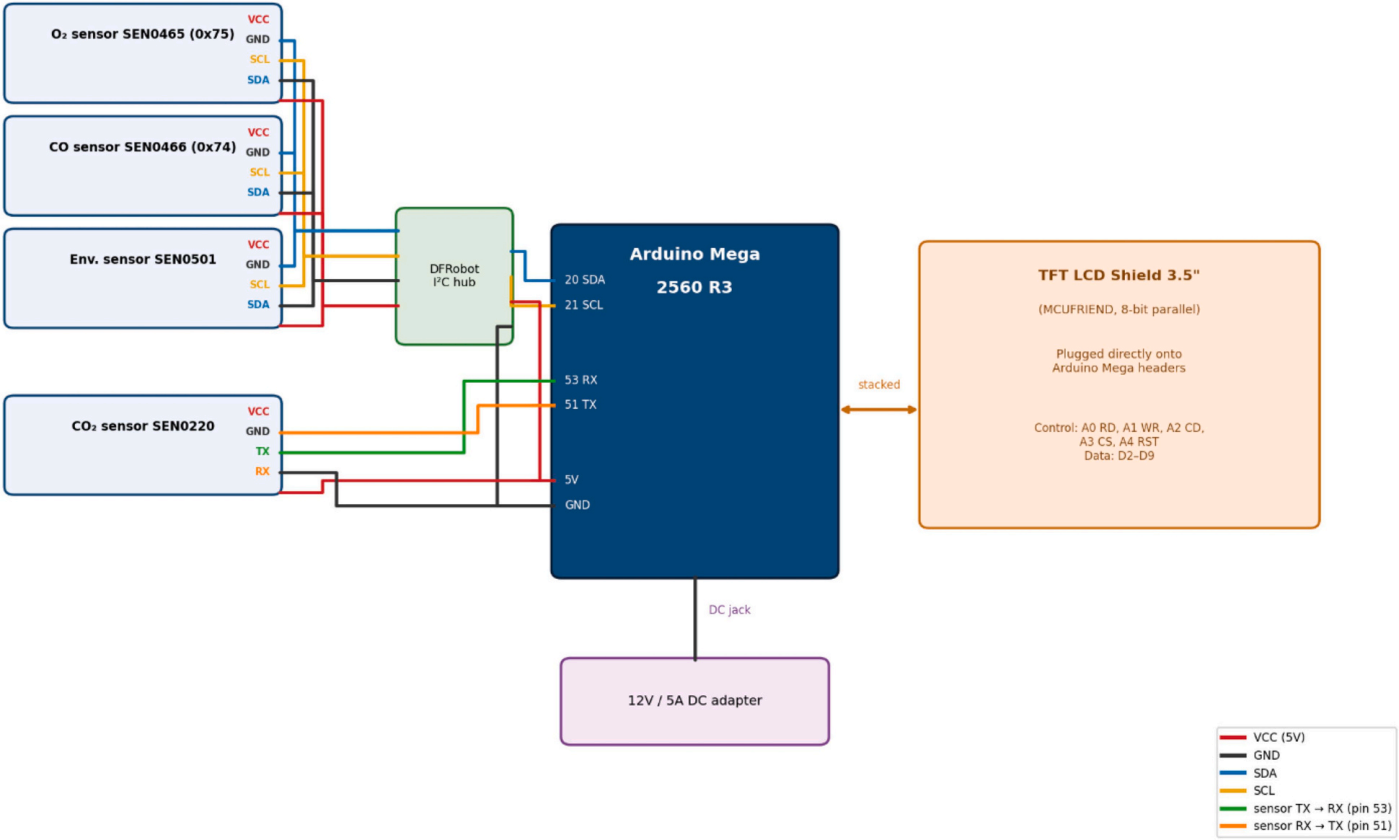
Wiring diagram of the ADESTI system showing the pin level connections and wire assignments between the sensors, the Arduino Mega 2560 R3, and the TFT display.

Fig. 2 shows the wiring diagram of the ADESTI system with the pin assignments of each module. The oxygen sensor, the carbon monoxide sensor, and the environmental sensor connect to the DFRobot I^2^C hub, which links to the I^2^C bus of the Arduino Mega 2560 R3 on pin 20 for SDA and pin 21 for SCL. The oxygen sensor uses address 0x75 and the carbon monoxide sensor uses address 0x74. The carbon dioxide sensor connects through the UART interface, with its transmit line to pin 53 and its receive line to pin 51. The TFT display is a 3.5 in. MCUFRIEND shield that plugs directly onto the Arduino Mega headers. It uses the control pins A0 to A4 and the data pins D2 to D9. The whole system is powered by a 12 V and 5 A DC adapter. The wire colors follow the legend for VCC, GND, SDA, SCL, and the UART lines.

**Fig. 3:**
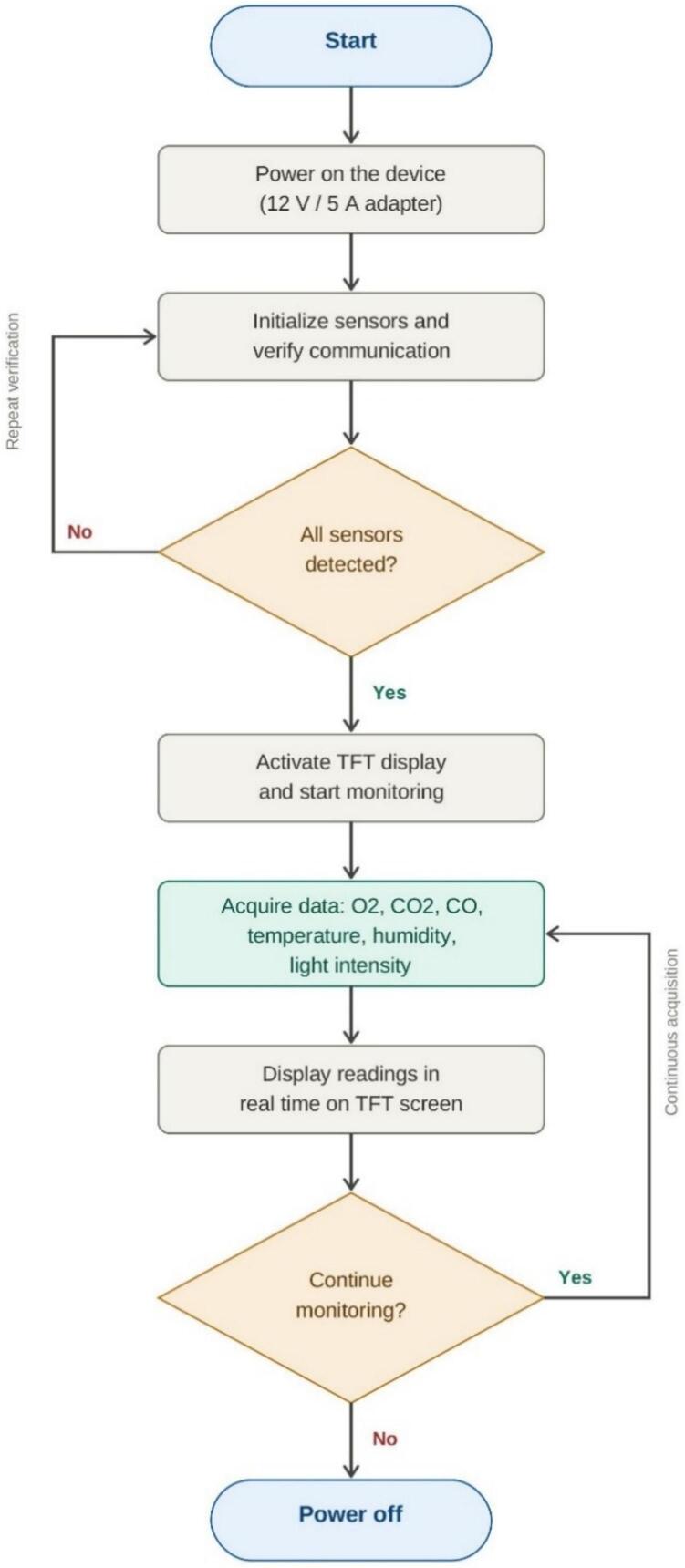
Flowchart of the ADESTI operation process, from power-on and sensor initialization to data acquisition and display.

After all hardware connections are completed, the firmware developed in the Arduino IDE is uploaded into the Arduino Mega 2560 R3 through a USB interface. The work flow of the device's firmware showed in Fig. 3 includes sensor initialization, data polling, display visualization, and safety threshold logic for continuous monitoring applications. Configuration parameters such as the serial baud rate and the display update interval can be modified in the firmware before upload. The current firmware version displays real time readings without automatic threshold logic.

If the display shows a sensor initialization failure, the wiring and the I^2^C address of the affected sensor should be checked, since the firmware waits at initialization until each sensor responds. If two I^2^C sensors do not respond, an address conflict should be checked, as each sensor must use a unique address, namely 0x75 for oxygen and 0x74 for carbon monoxide. If the carbon dioxide reading does not appear, the UART wiring on pins 53 and 51 should be verified.

**Fig. 4:**
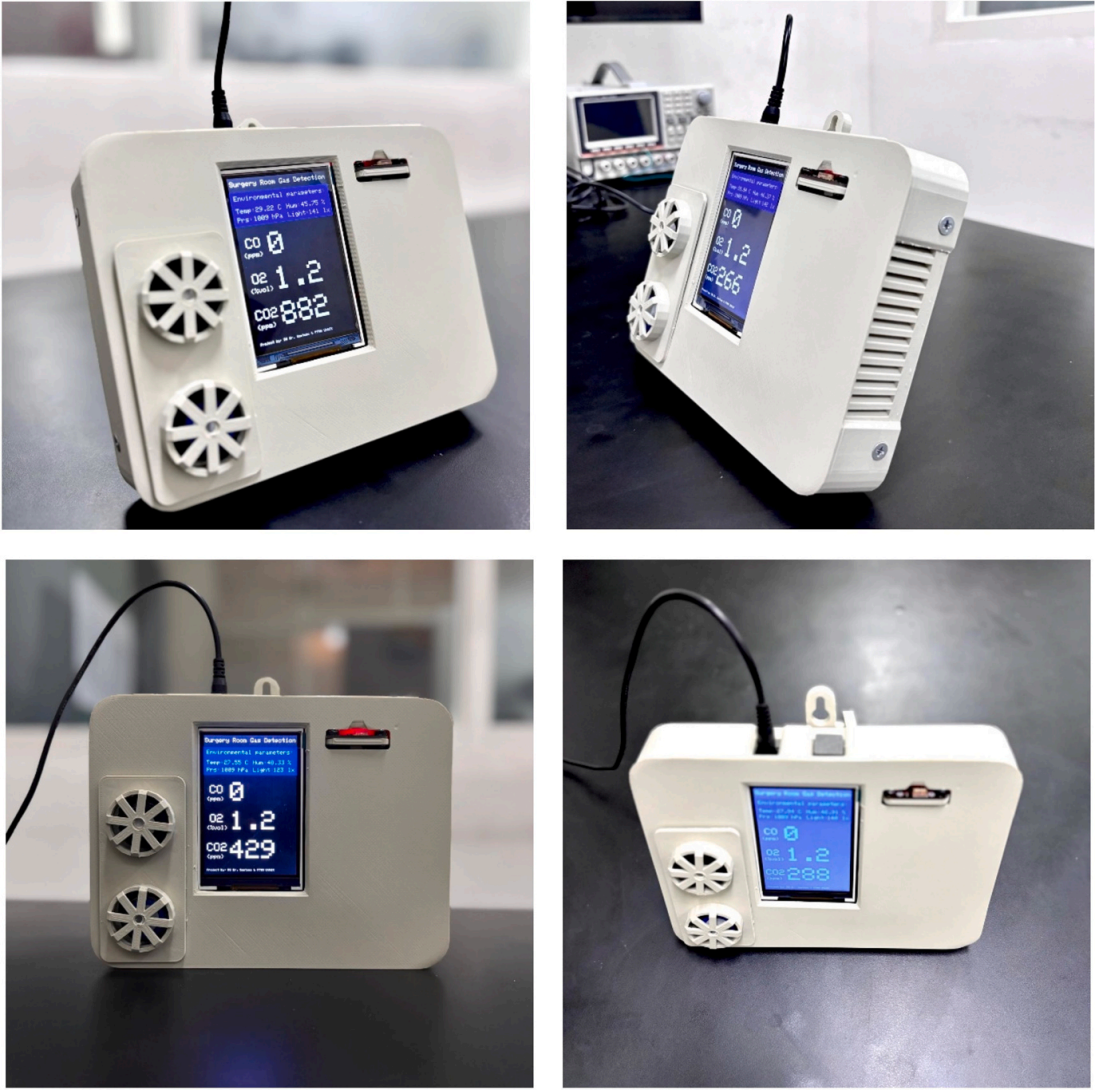
Operation of the ADESTI device showing the TFT display with real-time readings of gas concentrations and environmental parameters.

Showed in Fig. 4, the enclosure was fabricated from matte PLA + filament using a Bambu Lab A1 printer with Bambu Studio as the slicer. The parts were printed with a layer height of 0.2 mm, an infill density of 80 percent, and a horizontal print orientation. The nozzle temperature was set to about 220 °C and the bed temperature to about 75 °C. The enclosure consists of three parts, with a total print time of approximately 14 h. The enclosure design includes dedicated mounting spaces for sensors, ventilation openings for gas exposure, cable routing channels, and protective housing for the electronic components. Ventilation placement is an important design consideration because insufficient airflow can reduce gas sensor responsiveness and measurement accuracy. The modular casing also allows rapid maintenance and sensor replacement without disassembling the entire system. After the enclosure printing process is completed, all electronic modules are mounted inside the enclosure using eight M3 flat head screws of 6 mm length, with adhesive supports where necessary. Finally, the fully assembled system undergoes functional verification by powering the instrument and confirming that all sensors, display visualization, and data acquisition processes operate correctly. After assembly and before field deployment, the gas sensors should be calibrated against certified reference instruments, as described in Section 7.

Several design alternatives were evaluated during the development process. The ESP32 was initially considered for its integrated wireless communication capability. The Arduino Mega 2560 R3 was ultimately selected because it provided more stable multi-sensor communication, additional GPIO pins, and easier integration with multiple UART and I^2^C devices. A custom printed circuit board was also evaluated during the early design stage, but modular sensor modules were preferred to reduce fabrication cost, simplify replacement procedures, and accelerate prototyping. For enclosure fabrication, both ABS and PLA + were considered. PLA + was selected because it offers easier 3D printing implementation, better dimensional stability, lower warping tendency, and reduced manufacturing cost compared to ABS.

Safety precautions must be considered during both the construction and operation of the ADESTI system. All electronic assembly procedures should be performed while the power supply is disconnected to prevent electrical short circuits and accidental component damage. The 12 V/5 A power adapter must be operated according to its rated specifications and should not be exposed to excessive heat or moisture. During sensor calibration and gas exposure testing, the device should only be used in well-ventilated laboratory environments to avoid unintended accumulation of hazardous gases. Direct contact between liquids and electronic components should be avoided, because moisture may damage the sensors and cause unstable measurements. The electrochemical and NDIR gas sensors are intended for environmental monitoring only and should not be exposed to corrosive chemicals or to gas concentrations beyond their specified range. Periodic recalibration using certified reference instruments is recommended to maintain long-term measurement reliability. The developed hardware is intended solely for research and environmental monitoring applications and should not be considered a certified medical diagnostic or life-support instrument for clinical decision-making. The complete construction procedure is presented in the following steps.

• Prepare all electronic components: Arduino Mega 2560 R3 microcontroller, electrochemical O_2_ sensor, electrochemical CO sensor, NDIR CO_2_ sensor (SEN0220), SHTC3 temperature and humidity sensor, VEML7700 ambient light sensor, LCD display, DFRobot I^2^C hub, jumper cables, and a 12 V/5 A power adapter.
• Characterize each sensor individually before full integration to determine its actual specifications. Test each sensor using the Arduino IDE and compare its readings against certified reference instruments to obtain accuracy, precision, linearity, and measurement error. This characterization verifies operational functionality and communication compatibility while establishing the actual performance specifications of each sensor.
• Connect the gas and environmental sensors to the DFRobot I^2^C hub and UART interfaces. The CO_2_ sensor uses UART serial communication. The SHTC3 and VEML7700 sensors share the I^2^C bus with unique addresses to avoid communication conflicts.
• Connect the LCD display to the microcontroller for real-time visualization of sensor outputs. Arrange the wiring with proper cable management to minimize electrical noise.
• Upload the firmware (ADESTI_Firmware.ino) to the Arduino Mega 2560 R3 through the USB interface using the Arduino IDE.
• Design the 3D enclosure using SketchUp, with mounting positions and seats adapted to each component. The design should include sensor mounting spaces, ventilation openings aligned with the gas sensors, and cable routing channels. Export the finished model to STL format and fabricate the enclosure by 3D printing in PLA + material using fused deposition modeling.
• Mount all electronic modules inside the enclosure using eight M3 flat head screws of 6 mm length, with adhesive supports where necessary. The gas sensors should be mounted so that their sensing openings face the ventilation holes, ensuring direct airflow exposure, with a minimum clearance around each sensor.
• Power the assembled system with the 12 V/5 A adapter and verify that all sensors, the LCD display, and the data acquisition process operate correctly.
• Calibrate the gas sensors against certified reference instruments before measurement. Accuracy and precision can be validated by comparing readings with standard instruments under controlled conditions.
• Operate the device in the target environment, allowing continuous multi-sensor data acquisition with readings displayed in real time on the LCD.

## 6 Operation instructions

The operation of ADESTI begins by placing the device in a stable position within the operating room or laboratory environment where gas monitoring is required. Before powering the system, users must ensure that all sensors and wiring connections are securely attached and that the ventilation openings are unobstructed to allow proper gas circulation. The device is powered using a 12 V/5 A AC-to-DC adapter connected to the Arduino Mega 2560 R3 system. Once powered on, the firmware automatically initializes all sensors, verifies communication protocols, and activates the TFT display. After power on, the gas sensors require a short warm up period before the readings become reliable. The NDIR carbon dioxide sensor in particular requires a warm up of about 5 to 7 min. Before the warm up is complete, the carbon dioxide reading may fall outside the valid range or show unstable values. Users should therefore allow the device to run for several minutes after initialization before recording measurements. If any sensor connection fails during initialization, the system repeats the verification process until all sensors are successfully detected and operational.

After successful initialization, the device continuously acquires environmental data, including oxygen concentration, carbon dioxide concentration, carbon monoxide concentration, temperature, humidity, and ambient light intensity. All measured parameters are displayed in real time on the TFT display, enabling users to monitor operating room conditions directly without requiring external software. In the current version, the measured values are displayed in real time and are not automatically stored in internal memory. For data recording, the readings can be streamed to a connected computer through the serial interface at 115,200 baud, where they can be logged using the Arduino IDE serial monitor or an equivalent terminal application. Onboard data logging and wireless transmission are not implemented in the current version, as noted in the limitations. The firmware performs periodic data polling to maintain stable acquisition performance during long-duration operation. Measurements can be conducted continuously for several hours or days depending on monitoring requirements. For field applications in operating rooms, the instrument should be positioned near zones where air circulation needs to be monitored. It should be placed so that it does not interfere with the sterile field or the movement of the surgical team. The current firmware version displays real time readings without automatic threshold or alarm logic, so the interpretation of safe ranges is performed by the operator. The normal ambient oxygen concentration, measured with the reference instrument under controlled laboratory conditions, is about 20.9 percent by volume. For carbon dioxide and carbon monoxide, safety reference thresholds are used instead of measured values, since their concentrations in clean room air are very low. The commonly used occupational exposure references are about 5000 ppm for carbon dioxide and about 25 ppm for carbon monoxide, expressed as time weighted averages. These reference values can guide the operator when interpreting the readings. They can also be used by other researchers who wish to add threshold logic to the open source firmware.

During operation, users should periodically inspect the TFT display readings to ensure that all parameters remain within safe ranges. Elevated CO_2_ concentrations or abnormal gas fluctuations may indicate insufficient ventilation or air quality degradation in the room. If unstable readings occur, users should inspect sensor wiring, ventilation openings, and power supply stability before restarting the system. The device must not be exposed to liquids, excessive heat, or direct contamination from chemicals because these conditions may damage the sensors and reduce measurement accuracy. In addition, the gas sensors should only be operated in well-controlled environments to prevent sensor degradation caused by prolonged exposure to corrosive gases or excessive humidity. After operation is completed, the device can be safely powered off by disconnecting the external power adapter. The detailed operation procedure is presented in the following steps.

• Place the ADESTI device on a stable, flat surface within the operating room or laboratory environment where gas monitoring is required, ensuring that the ventilation openings are unobstructed to allow proper gas circulation.
• Connect the 12 V/5 A AC-to-DC adapter to the Arduino Mega 2560 R3 and to the power source to switch the device on.
• Allow the firmware to automatically initialize all sensors, verify the communication protocols, and activate the TFT display. If a sensor fails to connect, the system repeats the verification until all sensors are detected.
• After successful initialization, observe the real-time readings on the TFT display, which shows oxygen, carbon dioxide, and carbon monoxide concentrations together with temperature, humidity, and ambient light intensity.
• Position the instrument near zones where air circulation needs to be monitored, while keeping it clear of the sterile field and the movement of the surgical team.
• Periodically inspect the TFT display during operation to confirm that all parameters remain within safe ranges. Elevated CO_2_ concentrations or abnormal fluctuations may indicate insufficient ventilation or air quality degradation.
• If unstable readings occur, inspect the ventilation openings and power supply stability, then restart the device.
• After monitoring is complete, power off the device by disconnecting the external power adapter.
• Safety hazards. Keep the device away from liquids, excessive heat, and chemical contamination, as these may damage the sensors and reduce measurement accuracy. Operate the gas sensors only in well-controlled environments, since prolonged exposure to corrosive gases or excessive humidity causes sensor degradation. Operate the 12 V/5 A adapter strictly within its rated specifications.
• The device should be operated within the working range of its sensors, namely an ambient temperature between 0 and 50 °C and a relative humidity between 0 and 95 percent. Operation outside these ranges may reduce measurement accuracy.

## 7 Validation and characterization

The validation and characterization of the ADESTI system were conducted through both laboratory-scale testing and real-world field implementation in operating room environments. The primary objective of the validation process was to evaluate the stability, responsiveness, accuracy, precision, and long-term operational reliability of the developed environmental monitoring platform. The system was specifically designed for continuous monitoring of operating room air quality and environmental conditions within medical operating theatres. Therefore, the characterization process focused on evaluating the capability of the embedded system to acquire multi-sensor data continuously under varying environmental conditions and medical activities. A relevant use case of the ADESTI system is continuous environmental monitoring in operating rooms during surgical procedures involving anesthetic gases. During field implementation at Dr. Soetomo General Academic Hospital Surabaya, the system was deployed to monitor gas concentration fluctuations before, during, and after several medical procedures, including neural injection procedures, gallbladder surgery using sevoflurane and oxygen, and kidney surgery. The system successfully detected variations in O_2_ and CO_2_ concentrations associated with occupancy levels, ventilation conditions, and anesthetic usage throughout the surgical process.

### 7.1 Controlled environment measurement analysis

A laboratory-scale test was conducted to assess the performance of the gas and environmental-parameter-measuring instrument under controlled conditions prior to field deployment. The objective of the test was to evaluate accuracy, sensitivity, and stability across various conditions by measuring oxygen (O_2_), carbon dioxide (CO_2_), temperature, humidity, and ambient light. Calibration with standard gases was employed to ensure measurement precision, while the data collected contributed to refining the instrument’s design, optimizing data processing, and validating its technical specifications. The test was conducted on December 28, 2024, at the Laboratory of Control Systems at Universitas Airlangga. Conducted by research assistants, the test duration was 24 h, with measurements taken at 30-minute intervals.

**Fig. 5:**
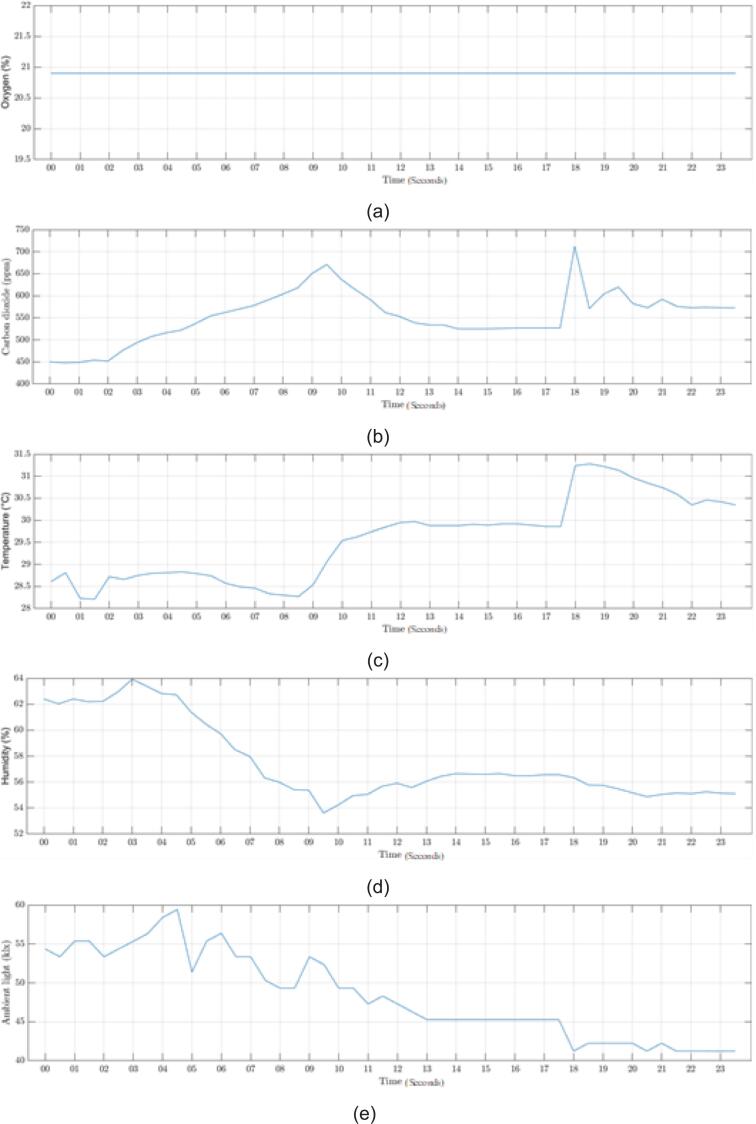
Graphs of environmental parameter changes for: (a) O_2_, (b) CO_2_, (c) temperature, (d) humidity, and (e) ambient light.

The results showed in Fig. 5 indicated that oxygen (O_2_) concentration remained stable at approximately 21% throughout the day, as illustrated in Fig. 5(a), reflecting normal atmospheric conditions with no significant environmental disturbances. Meanwhile, as shown in Fig. 5(b), carbon dioxide (CO_2_) levels fluctuated, starting at 450 ppm in the early morning, suggesting good ventilation and minimal human activity. The concentration peaked at 660 ppm by 8 a.m. due to increased occupancy, dropped to 475 ppm around midday, and rose again to 575 ppm in the afternoon. The highest peak, nearly 700 ppm, occurred at 8p.m., likely influenced by factors namely crowd density, CO_2_-emitting devices, and reduced ventilation, before gradually declining. However, carbon monoxide (CO) levels remained at zero throughout the test, as the laboratory was isolated from combustion engine sources, confirming a clean air environment.

Temperature and humidity levels exhibited anticipated variations, as demonstrated in Fig. 5(c) and Fig. 5(d), respectively. The temperature commenced at 26.5°C, decreased to 25°C during the early morning, reached a peak of 31°C at midday, and then gradually declined towards the end of the day. Humidity was initially above 60%, reduced to 52% prior to midday, and subsequently stabilized around 55%, signifying a transition to drier conditions. Ambient light intensity demonstrated more dynamic fluctuations, as depicted in Fig. 5(e), starting at a moderate level, reaching a maximum of 70 klx, then decreasing gradually with a minor increase in the afternoon before stabilizing at low levels in the evening.

### 7.2 Sensor accuracy and precision analysis

The sensor's accuracy is assessed by comparing its measurements with those of a standard instrument known for its high reliability and precision. The sensor under evaluation is positioned within similar measuring conditions as the standard instrument, which also functions as an accurately calibrated reference or benchmark. The parameter being measured, or the concentration level, is tested at various levels to evaluate how closely the sensor's readings match the true values. The discrepancy between the results from the sensor and the standard instrument is analyzed to determine the sensor's accuracy level.

**Fig. 6:**
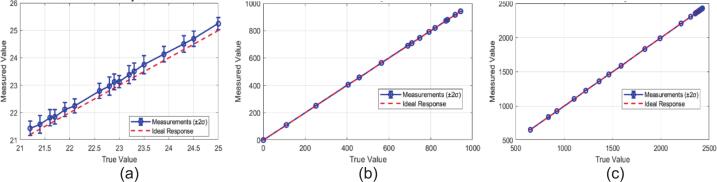
Error measurement results on each sensor (tested instrument) which is shown by its measured value; (a) O_2_ sensor, (b) CO_2_ sensor, and (c) CO sensor.

The measurement accuracy and precision relative to the true value depicted in Fig. 6 are commonly employed in the validation of sensors or other measuring devices. The true value is then compared to the measured value. The red dotted line indicates the ideal response, representing a scenario where the measured value consistently equals the true value. Conversely, the blue dots denote the measured values at each data point. Furthermore, the blue lines with error bars illustrate the measured values with a confidence interval of ± 2σ (twice the standard deviation), which reflects the measurement's precision. The compatibility between the red dotted line (true value) and the blue line (measured value) signifies the accuracy of the measurement.

The calculated value depicted in Fig. 6(a) indicates that the O_2_ sensor has an absolute error of 0.29%, a standard deviation of 0.14%, and an RMSE of 0.49. Furthermore, based on Fig. 6(b), the calculated results indicate that the CO_2_ sensor has an absolute error of 1.16% and a standard deviation of 0.15%, with an RMSE of 5.19. Lastly, the calculated values from Fig. 6(c) indicate that the CO sensor has an absolute error of 6.21% and a standard deviation of 0.14%, with an RMSE value of 28.20.

Measurement uncertainty was considered during the monitoring process. For each sensor, the uncertainty was quantified through the standard deviation and the root mean square error obtained from the paired comparison against the reference instrument. The oxygen sensor showed a standard deviation of 0.14% and an RMSE of 0.49. The carbon dioxide sensor showed a standard deviation of 0.15% and an RMSE of 5.19. The carbon monoxide sensor showed a standard deviation of 0.14% and an RMSE of 28.20. These low standard deviation values indicate a small measurement uncertainty and stable sensor readings throughout the monitoring period.

Table 5 presents the accuracy test results for various sensors, comparing the measured outcomes with those of standard instruments. The tests encompass six parameters: O_2_, CO_2_, CO, temperature, humidity, and ambient light. The accuracy of each sensor is assessed based on the mean absolute error, which is subsequently utilized to determine the percentage of accuracy. Additionally, the precision of each sensor is evaluated using the standard deviation values. The results denote that all tested sensors deliver satisfactory performance and reliability for their respective parameters, although some require particular attention.

**Table 5:** Accuracy test method with a standard instrument.

**Tested Parameters**	**Tested Instruments**	**Standard Instruments**	**Test Range**	**Accuracy (%)**	**Precision (%)**
O_2_	SEN0465	Lutron PO_2_-250	0–25%Vol	99.71	99.86
CO_2_	SEN0220	Lutron GC-2028	0–50000 ppm	98.84	99.85
CO	SEN0466	Lutron GCO-2008	0–1000 ppm	93.79	99.86
Temperature	SHTC3	Lutron MHB-382SD	−40 – 125°C	95.42	91.23
Humidity	SHTC3	Lutron MHB-382SD	0–100%	97.13	93.74
Ambient Light	VEML7700	Lutron UV-3404	0–120 klx	91.46	94.65

A direct quantitative comparison can be drawn between ADESTI and the dedicated commercial instruments used as benchmarks. The manufacturer specified accuracy of each reference instrument is as follows. The Lutron PO_2_-250 has an accuracy of ±(1% reading + 0.2% O_2_). The Lutron GC-2028 has an accuracy of ±40 ppm up to 1000 ppm, and ±5% reading from 1000 to 3000 ppm. The Lutron GCO-2008 has an accuracy of ±(5% reading + 2 ppm). The Lutron MHB-382SD has an accuracy of ±0.8 °C for temperature, and ±(4% reading + 1% RH) at or above 70% RH.

For every parameter, the measured accuracy of ADESTI falls within or close to the manufacturer tolerance band of its corresponding Lutron reference instrument. This shows that the integrated low cost platform reaches measurement performance comparable to dedicated single parameter meters. The comparison also underlines a practical benefit of ADESTI. Covering the same set of parameters with commercial instruments would require four separate Lutron meters. ADESTI combines all of them into one device. It also adds ambient light sensing, which none of the four Lutron instruments provides.

**Fig. 7:**
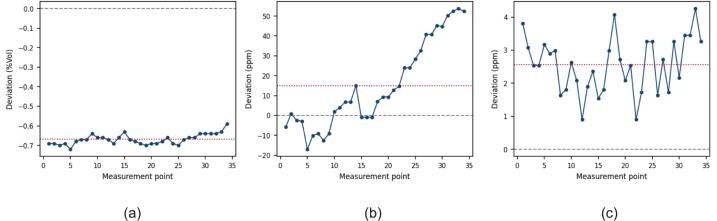
Deviation between the ADESTI sensor readings and the reference instrument at each measurement point for: (a) O_2_ sensor, (b) CO_2_ sensor, and (c) CO sensor.

Fig. 7 shows the deviation between the ADESTI sensor readings and the reference instrument at each measurement point. The deviation is defined as the sensor reading minus the reference reading. For the oxygen sensor in Fig. 7(a), the deviation remains small and stable around a mean of about minus 0.67 %Vol, with a very low spread. This indicates a consistent and repeatable measurement. For the carbon dioxide sensor in Fig. 7(b), the deviation is larger and varies across the measurement range, with a mean of about plus 15 ppm. For the carbon monoxide sensor in Fig. 7(c), the deviation shows a larger and consistent positive offset relative to the reference. This larger deviation reflects the known limitation of the low cost electrochemical carbon monoxide sensor, as acknowledged in Section 1.3. In the operating room environment, the carbon monoxide concentration remained at or near zero, so this offset has limited impact on the intended monitoring application.

### 7.3 Sensor linearity analysis

This test evaluates sensor response to changes in gas concentration, ensuring a consistent linear relationship across its measurement range. Sensors were tested at varying gas concentrations, from low to high, and their outputs were compared with reference values from a standard instrument. The linearity test is essential for confirming the sensors’ reliability under different operating conditions, particularly in medical applications.

**Fig. 8:**
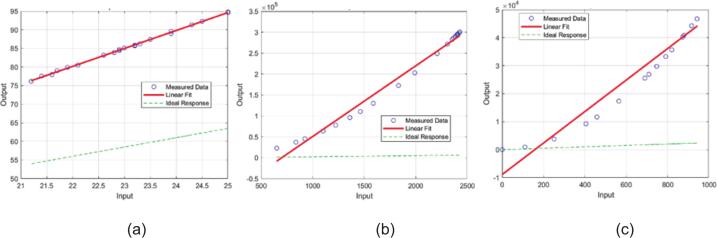
Linearity test results on each sensor reading for: (a) O_2_, (b) CO_2_, and (c) CO.

As illustrated in Fig. 8(a), the sensitivity of the O_2_ sensor is quantified at 4.8198 units per incremental input. The noteworthy R^2^ value of 0.9987 signifies an excellent linear fit, with merely 1.90% non-linearity, thereby confirming minimal deviation and high measurement precision. Fig. 8(b) depicts a robust linear correlation between the input and output of the CO_2_ sensor. The green dotted line indicates the ideal response, with a measured sensitivity of 169.0307. Although there is a slight deviation from this ideal value, the R^2^ of 0.9852 demonstrates that the model fits the data well, despite a non-linearity of 11.16%. Fig. 8(c) shows the CO sensor response, with a measured sensitivity of 56.2568. The R^2^ value of 0.9518 suggests that the linear model effectively accounts for the variability in output. However, the non-linearity of 19.02% reflects a more considerable deviation relative to the other sensors. Overall, the findings indicate that the sensors possess strong linearity with only minor deviations. These results confirm their appropriateness for dependable anesthetic gas monitoring within medical settings.

### 7.4 Field measurement and data acquisition

The measured parameters are interpreted with reference to established indoor air quality and occupational safety guidelines. Oxygen concentration is considered safe within the range of 19.5% to 23.5% according to OSHA respiratory protection standards. Carbon dioxide concentration in occupied spaces is recommended to remain below 1000 ppm based on ASHRAE Standard 62.1 for ventilation of buildings. Carbon monoxide should remain below 9 ppm for an 8-hour time-weighted average according to WHO indoor air quality guidelines. The measurement of operating room air quality is conducted at a strategic location within the operating room to ensure precise and representative data collection. Gas concentration measurements obtained during the surgical procedure are recorded at five-minute intervals. Data acquisition is performed multiple times at three distinct stages: preoperative, intraoperative, and postoperative, providing a comprehensive depiction of operating room air composition throughout the medical procedure. The data acquired in the operating room on 29th April, 30th April, and 30th May 2024 are presented in Table 6. Based on the information in Table 6 and Table 7, it is observed that, generally, temperature and CO_2_ levels decrease, while minor fluctuations occur in O_2_ levels.

**Table 6:** Gas concentration sample data statistics based on the date of data acquisition.

**Date**	**Statistics**	**Temperature (°C)**	**O_2_ (%)**	**CO (ppm)**	**CO_2_ (ppm)**
Monday, 29th April 2024	Mean MedianMode	21.46 20.920.3	17.15 15.615.5	0.04 00	607.52 605.598
Tuesday, 30th April 2024	Mean MedianMode	16.92 16.716.7	15.66 15.715.7	0.05 00	619.14 607.571
Friday, 30th May 2024	Mean MedianMode	21.97 21.6521.3	15.61 15.615.6	0.07 00	603.85 605.567

**Table 7:** Gas concentration sample data statistics based on the types of procedure in the operating room.

**Types of Procedure**	**Mean Parameter Value**			
**& Gases Used**	**Temperature (°C)**	**O_2_ (%)**	**CO (ppm)**	**CO_2_ (ppm)**
Neural Injection Without Gas				
Preoperative	25.2	15.9	0.7	620.0
Intraoperative	21.5	18.5	0.0	602.4
Postoperative	21.2	15.6	0.0	588.2
Neural Injection with desflurane				
Preoperative	21.8	15.6	0.0	594.0
Intraoperative	20.2	15.5	0.0	603.9
Postoperative	21.9	15.5	0.0	665.0
Gallbladder Removal with sevoflurane & oxygen				
Preoperative	18.3	15.8	0.3	580.7
Intraoperative	16.6	15.7	0.0	643.1
Postoperative	16.7	15.4	0.0	558.1
Kidney Surgery				
Preoperative	23.9	15.7	0.6	632.7
Intraoperative	21.8	15.6	0.0	605.1
Postoperative	20.6	15.6	0.0	561.3

Additionally, the data categorized according to the types of procedures conducted in the operating room are shown in Table 7. The instrument measured all three target gases, O_2_, CO_2_, and CO, throughout the field deployment. However, the CO readings remained at or below the detection threshold of the sensor during all measurement sessions in the operating room. This result is consistent with the absence of combustion sources in this clinical environment. For clarity, the field measurement tables therefore report O_2_ and CO_2_ only, while CO is summarized separately as below detection.

**Fig. 9:**
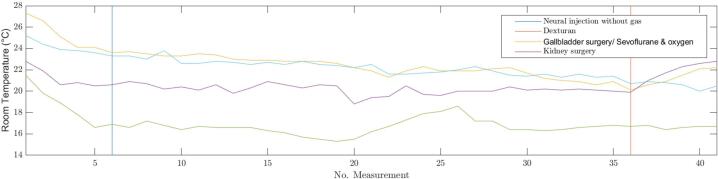
Operating room temperature changes on four different surgeries.

The changes in operating room temperature, as depicted in Fig. 9. They are derived from four distinct medical procedures: neural injection without gas, neural injection with desflurane, gall bladder surgery utilizing sevoflurane and oxygen, and kidney surgery. The temperature in the operating room generally decreases during these procedures, with varying patterns observed in each case. Specifically, the temperature consistently declines from approximately 25°C to around 21°C during the neural injection without gas procedure. During desflurane administration, temperature fluctuations are minimal, maintaining a relatively stable level near 22°C. In the case of gall bladder surgery with sevoflurane and oxygen, there is a notable drop in temperature from 18°C to 16°C. Although fluctuations occur during kidney surgery, the temperature ultimately decreases from 24°C to 21°C. The vertical blue line indicates a significant moment in the procedure, namely the commencement of a specific phase, while the vertical red line marks the conclusion of that phase. The graph infers that each type of medical procedure influences room temperature in distinct patterns.

**Fig. 10:**
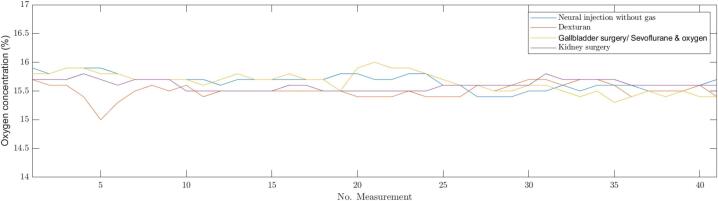
Oxygen concentration level changes during four different surgeries.

The oxygen concentration levels tend to remain stable within the range of approximately 15.5% to 16%, as depicted in Fig. 10, with several minor fluctuations observed during each procedure. The neural injection procedure conducted without the use of gas (represented by the blue line) maintains a stable oxygen level, approximately 15.6%. Additionally, the same procedure incorporating desflurane (depicted by the orange line) exhibits negligible fluctuations, with an average concentration of about 15.5%. A similar pattern of minor fluctuations is observed during gallbladder surgery with sevoflurane and oxygen (illustrated by the yellow line). Conversely, the oxygen level during kidney surgery (indicated by the purple line) remains relatively stable, with slight variations around 15.7%. The fluctuations observed on the graph may be attributed to procedural variations or the specific use of medical instruments, which may influence oxygen levels in the environment. The measured oxygen concentrations were consistently lower than the typical ambient level of 20.9%, ranging between approximately 15.5% and 16% across the observed procedures. Although the absolute readings differ from atmospheric values, the relative variation across the operating room and the surgical procedures still reflects changes in air composition and ventilation. This persistent offset is treated as a limitation of the field measurement and is discussed in the following paragraph.

**Fig. 11:**
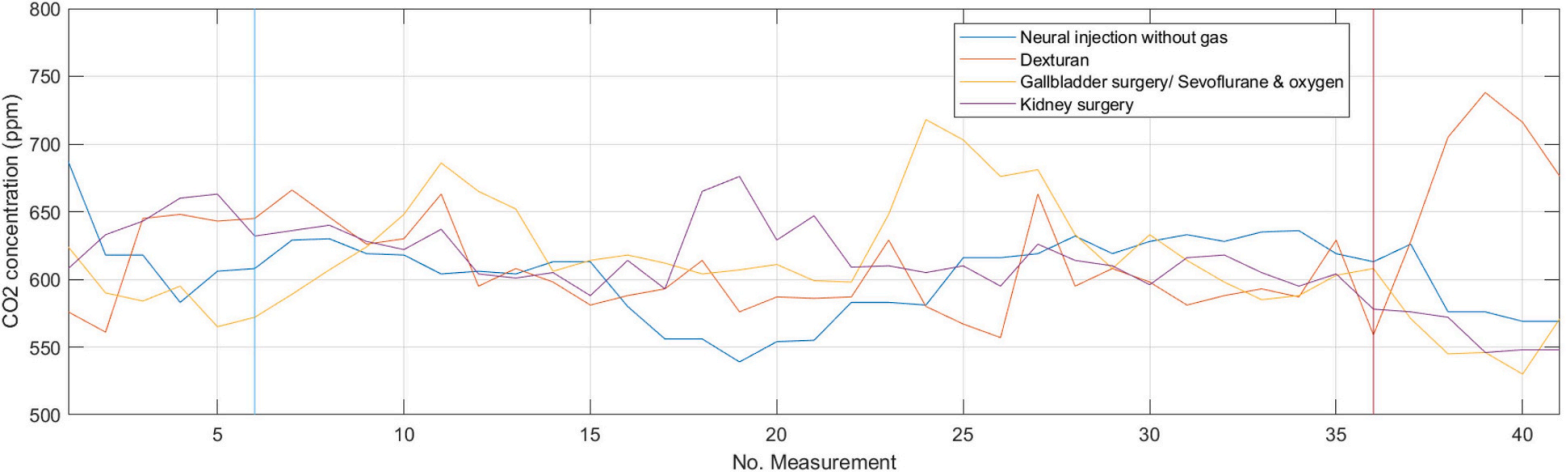
CO_2_ concentration level changes during four different surgeries.

Fluctuations in carbon dioxide (CO_2_) concentration from four medical procedures are illustrated in Fig. 11, which shows an overall range of 550–800 ppm, with variations in each instance. The CO_2_ level during neural injection without gas tends to be lower and more stable, remaining around 550–600 ppm, with a minor decrease observed at the conclusion of the measurement. The similar procedure using desflurane exhibits greater fluctuations, with one of the peaks approaching approximately 750 ppm. Conversely, gallbladder surgery involving gases namely sevoflurane and oxygen maintains relatively stable concentrations, generally fluctuating between 550 and 750 ppm, despite a notable increase to 800 ppm during a specific phase. Kidney surgery exhibits significant fluctuations within the 550–750 ppm range, with several peaks occurring mid-measurement. These values remain below the indoor air quality guideline of 1000 ppm commonly cited for occupied spaces, indicating that ventilation in the operating rooms was generally adequate during the observed procedures, despite occasional elevations. It is important to note that the carbon monoxide (CO) concentration in the operating room remains at zero, as the room must retain sterility. Consequently, CO levels are consistently recorded as zero.

The persistent offset in O_2_ concentration relative to the laboratory test, in which the sensor measured 20.9% under normal atmospheric conditions, may be attributed to several factors. These include the proximity of the sensor inlet to localized airflow from the operating room ventilation system, sensor drift under the cooler operating room temperature, and the absence of an on-site recalibration before each session. Future deployments will include a pre-session calibration step and a controlled study of sensor placement to address this offset.

## Ethics statements

This study involved environmental gas measurements conducted in the operating rooms of Dr. Soetomo General Academic Hospital Surabaya, Indonesia. The instrument measured only ambient air parameters and did not collect any patient data or interact with patients directly. The study was approved by the ethics committee of General Academic Hospital Dr. Soetomo, Surabaya (approval number: 1258/KEPK/III/2025. As the study did not involve human subjects or the collection of identifiable personal data, individual informed consent was not required; institutional permission to conduct the in-room measurements during surgical procedures was obtained from the hospital.

## CRediT authorship contribution statement

**Prisma Megantoro:** Writing – review & editing, Validation, Supervision, Software, Methodology, Investigation, Formal analysis, Data curation, Conceptualization. **Anna Surgean Veterini:** Writing – review & editing, Supervision, Resources, Project administration, Funding acquisition. **Nagesparan Ainarappan:** Writing – review & editing, Validation, Supervision. **Nayu Nurrohma Hidayat:** Writing – original draft, Validation. **Fikarul Mujtahida:** Writing – original draft, Validation. **Mochammad Dava Septiadi Pratama:** Writing – original draft, Validation. **Muhammad Aqil Afham Rahmat:** Writing – review & editing.

## Declaration of competing interest

The authors declare that they have no known competing financial interests or personal relationships that could have appeared to influence the work reported in this paper.
